# Propolis Extracts Inhibit UV-Induced Photodamage in Human Experimental In Vitro Skin Models

**DOI:** 10.3390/antiox8050125

**Published:** 2019-05-09

**Authors:** Athanasios Karapetsas, Georgia-Persephoni Voulgaridou, Manolis Konialis, Ilias Tsochantaridis, Spyridon Kynigopoulos, Maria Lambropoulou, Maria-Ioanna Stavropoulou, Konstantina Stathopoulou, Nektarios Aligiannis, Petros Bozidis, Anna Goussia, Konstantinos Gardikis, Mihalis I. Panayiotidis, Aglaia Pappa

**Affiliations:** 1 Department of Molecular Biology & Genetics, Democritus University of Thrace, 68100 Alexandroupolis, Greece; karapetsas_than@yahoo.gr (A.K.); georgiavou_85@hotmail.com (G.-P.V.); manolis_konialis@yahoo.gr (M.K.); iliatsoc@gmail.com (I.T.); 2 Laboratory of Histology & Embryology, School of Medicine, Faculty of Health Sciences, Democritus University of Thrace, 68100 Alexandroupolis, Greece; spyroskinigopoulos@hotmail.com (S.K.); mlambro@med.duth.gr (M.L.); 3 Department of Pharmacy, Division of Pharmocognosy & Natural Products Chemistry, National and Kapodistrian University of Athens, 15771 Athens, Greece; mstavropoul@yahoo.gr (M.-I.S.); kstatho@pharm.uoa.gr (K.S.); aligiannis@pharm.uoa.gr (N.A.); 4 Department of Pathology, School of Health Sciences, University of Ioannina, 45110 Ioannina, Greece; pbozidis@cc.uoi.gr (P.B.); agoussia@uoi.gr (A.G.); 5 APIVITA SA, Industrial Park, Markopoulo, 19003 Athens, Greece; gardikis-k@apivita.com; 6 Department of Applied Sciences, Northumbria University, Newcastle Upon Tyne NE1 8ST, UK; m.panagiotidis@northumbria.ac.uk

**Keywords:** propolis, antioxidant, antiaging, photoprotective, DNA damage, matrix metalloproteinases, HaCaT, reconstituted skin

## Abstract

The aim of this study was to assess the antioxidant, photoprotective, and antiaging effects of Greek propolis. Propolis was subjected to n-heptane or methanol extraction. Total phenolic/flavonoid content and antioxidant potential were determined in the extracts. Promising extracts were evaluated for their cytoprotective properties using human immortalized keratinocyte (HaCaT) or reconstituted human skin tissue following exposure to UVB. Assessment of cytotoxicity, DNA damage, oxidative status, and gene/protein expression levels of various matrix metalloproteinases (MMPs) were performed. The propolis methanolic fractions exhibited higher total phenolic and flavonoid contents and significant in vitro antioxidant activity. Incubation of HaCaT cells with certain methanolic extracts significantly decreased the formation of DNA strand breaks following exposure to UVB and attenuated UVB-induced decrease in cell viability. The extracts had no remarkable effect on the total antioxidant status, but significantly lowered total protein carbonyl content used as a marker for protein oxidation in HaCaT cells. MMP-1, -3, -7, and -9, monitored as endpoints of antiaging efficacy, were significantly reduced by propolis following UVB exposure in a model of reconstituted skin tissue. In conclusion, propolis protects against the oxidative and photodamaging effects of UVB and could be further explored as a promising agent for developing natural antiaging strategies.

## 1. Introduction

Skin comprises the largest organ of the human body and the main barrier protecting the organism from various pathogens and environmental stressors, including ultraviolet radiation (UVR) [1]. UVR is distinguished into UVC (200–280nm), UVB (280–315nm), and UVA (315–400nm) [2]; UVC is absorbed by the stratosphere, while UVA and UVB are able to penetrate various layers of the skin [3]. UVB radiation causes a spectrum of DNA modifications, with the most prominent being the formation of cyclobutane-pyrimidine dimers (CPD) and 8-hydroxy-2′-deoxyguanosine adducts [4], which have been implicated in photo-induced carcinogenesis [5]. UVB radiation can also cause accumulation of reactive oxygen species (ROS) leading to generation of oxidative stress. To this end, ROS can react with a variety of biological macromolecules like proteins, DNA, and lipids, causing their oxidation [6]. Noteworthy, through ROS formation, UVB induces activator protein-1 (AP-1) overexpression along with the upregulation of collagen-degrading enzymes like matrix metalloproteinases (MMPs) [7]. Overall, UVB stimulates collagen degradation and inhibits procollagen biosynthesis resulting in loss of collagen content and wrinkle formation, thus inducing skin photoaging [8]. The adverse effects of UVB exposure can be potentially prevented by the use of natural products with photoprotective properties and so there is tremendous interest for their exploitation for antiaging product formulations [9].

Propolis is a mixture of resin, bee wax, pollen, and salivary gland secretions produced by honeybees, primarily for sealing their hives. It consists of polyphenols, flavonoids, terpenoids, aglycones, and phenolic acids and their esters [10]. Similarly to all honey bee products, its actual chemical composition varies and largely depends on the geographical region, the local vegetation, and the collection period [11]. Since ancient times, propolis has been used in wound healing as an antiseptic, while in recent years it has attracted even more attention due to its broad-range biological and pharmacological properties [12]. Thus, a plethora of studies have investigated the antioxidant [13,14,15,16,17,18], antimicrobial [19,20,21], anti-inflammatory [22,23,24,25], immune-modulatory and antitumor activities of propolis samples collected from different geographical regions [26,27,28,29,30]. Moreover, it has been shown that water, ethanol, and methanol extracts of propolis possess strong free radical scavenging activity. For instance, a study utilizing a topical treatment protocol based on propolis extracts (in mice pre- or post-UVB exposure) was shown to decrease malondialdehyde formation, which is an advanced oxidation product of lipid peroxidation and a recognized oxidative stress biomarker. [31]. In addition, it has been demonstrated that propolis extracts can interfere with viral replication and so can lead to a reduction of the viral titer [32]. Propolis has a very complex chemical composition, consisting of a wide range of compounds exerting a diverse range of pharmacological actions (e.g., antibacterial, anti-inflammatory, healing, anticarcinogenic, antifungal, antiviral, etc.). Of these, caffeic acid and quercetin have been long recognized for their ability to regulate the anti-inflammatory capacity of propolis by reducing production of eicosanoids and inhibiting the lipoxygenase pathway of arachidonic acid formation and thus modulating the inflammatory response [33]. Additionally, propolis extracts were shown to alter the expression profile of pro- and anti-inflammatory genes like TNFa, IFNγ, TLRs, and IL-1, -6, and -10, and modulate monocyte and lymphocyte recruitment [34,35,36]. Finally, several studies have reported the antiproliferative effect of propolis against a variety of human cancer cell lines [37,38,39,40] while it also appeared to decrease the expression of cyclins and promote apoptosis through the induction and/or inhibition of pro-apoptotic and anti-apoptotic genes, respectively [40,41,42].

However, still little is known regarding the protective potential of propolis against UVR-induced skin photoaging. Thus, the aim of the present study was to determine the antiaging potential of propolis after exposure to UVB. For this reason, propolis samples collected from different geographic regions of Greece were extracted by employing n-heptane and methanol extraction protocols. The resulting most promising propolis extracts were further evaluated for their (i) cytotoxicity profile, (ii) total antioxidant status (including in vitro activity levels), (iii) ability to inhibit free radical generation (measured as total protein carbonyl content), and (iv) suppression of photo-induced aging (monitored as inhibition of the UVB-induced upregulation of MMPs) in human experimental in vitro (immortalized keratinocytes and reconstituted tissue) skin models.

## 2. Materials and Methods

### 2.1. Propolis Samples Collection, Extraction and Processing

Ten propolis samples (PR_1–PR_10) were collected from different geographic regions of Greece during spring or fall season (Table 1). The samples were subjected to two-step sequential ultrasound-assisted extraction with n-heptane and methanol resulting into 10 *n*-heptane (PR_1a–PR_10a) and 10 methanolic (PR_1b–PR_10b) extracts, all of which were examined for their phenolic and flavonoid content. In addition, the methanolic extracts were submitted to a high-performance thin-layer chromatography (HPTLC) profiling and were also evaluated for their cell-free (in vitro) antioxidant activity, while the most promising extracts were further evaluated for their potential antimutagenic and antiaging properties.

### 2.2. Assessment of Total Phenolic Content

The total phenolic content of propolis extracts was determined by the Folin–Ciocalteu method [43,44]. In brief, 25 μL of propolis extract or standard solution of gallic acid (2.5, 5, 10, 12.5, 20, 25, 40, 50, 80, 100 μg/mL) in DMSO were added to 125 μL of a Folin–Ciocalteu solution (10%), followed by the addition of 100 μL of 7.5% sodium carbonate in a 96-well plate. The samples were incubated for 30 min, in darkness, at room temperature. Finally, the absorbance at 765 nm was measured using a TECAN Infinite m200 PRO multimode reader (Tecan Group, Männedorf, Switzerland). All measurements were performed in triplicate, the mean values were interpolated in a gallic acid calibration curve, and the total phenolic content was expressed as mg gallic acid equivalents (GAE) per gram of dry extract.

### 2.3. Assessment of Total Flavonoid Content

The total flavonoid content of the propolis extracts was determined by the aluminum chloride colorimetric assay as previously described [45]. Briefly, 50 μL of propolis extracts or standard solution of quercetin (2.5, 5, 10, 40, 80, 120, 160, 200 μg/mL) in DMSO were added to 20 μL of 10% of aluminum chloride solution and then mixed with 160 μL of 95% ethanol. Eighty percent (80%) ethanol was used as reagent blank. Finally, 20 μL of 1 M sodium acetate were added to the samples and incubated for 40 min, in darkness, at room temperature. The absorbance was measured at 415 nm with a TECAN Infinite m200 PRO multimode reader (Tecan Group, Männedorf, Switzerland). Total flavonoid contents were expressed as mg quercetin equivalents (QE) per gram of dry extract. All samples were analyzed in triplicate.

### 2.4. Evaluation of Cell-Free Antioxidant Activity by ABTS (2,2′-Azino-bis(3-Ethylbenzothiazoline-6-Sulfonic Acid) and DPPH (2,2-Diphenyl-1-Picrylhydrazyl) Assays

The radical scavenging activity of propolis extracts was estimated by the ABTS and the DPPH assays as previously described [46,47,48] with minor modifications. For the ABTS assay, the ABTS radical cation (ABTS•^+^) was produced through the reaction of 7 mM ABTS with 2.45 mM potassium persulfate, in dark, at room temperature for 12 h. The ABTS•^+^ solution was diluted in water to give an absorbance of 0.7 ± 0.01 at 734 nm. Then, the ABTS•^+^ solution (100 μL) was added to 50 μL of different extract concentrations (2–50 μg/mL) and incubated, at room temperature, for 10 min in the dark. Finally, the absorbance was measured at 734 nm in a TECAN Infinite m200 PRO multimode reader (Tecan Group, Männedorf, Switzerland). In each experiment, the tested sample in ethanol was used as blank and the ABTS•^+^ radical solution with H_2_O was used as control. The radical scavenging capacity of the sample was expressed as the percentage of ABTS^•+^ elimination calculated according to the following equation:

% Reduction = {[1−(A_Δ_−A_B_)]/A_T_}100, (1)

where A_Δ_ is the absorption of the sample, A_T_ the absorbance of control, and A_B_ the absorbance of the sample without the ABTS radical. Trolox was used as positive control (IC_50_ = 21.1 μg/mL).

For the DPPH assay, serial dilutions (1.25–5 mg/mL) of propolis extracts were prepared using dimethylsulfoxide (DMSO) as a solvent. Ten (10) μL of each sample were mixed with 190 μL of DPPH solution (12.4 mg/100 mL in ethanol) in a 96-well plate and then subsequently incubated, at room temperature, for 30 min in darkness. Finally, the absorbance was measured at 517 nm in a TECAN Infinite m200 PRO multimode reader (Tecan Group, Männedorf, Switzerland). All determinations were performed in triplicate. The % inhibition of the DPPH radical for each dilution was calculated using the following formula:

% Inhibition = {[1 − (A_Δ_ − A_B_)]/A_T_} × 100. (2)

Gallic acid was used as positive control (IC_50_ = 4.5 μg/mL). Based on the values derived from the % inhibition of the radical, reference curves were made for each extract, from which the IC_50_ values (μg/mL) were calculated (extract concentration − inhibition %).

### 2.5. HPTLC Profiling

For the chemical fingerprinting of the methanolic extracts, the samples were applied on HPTLC plate silica gel 60, 20 × 10 cm, as 8 mm bands, using an automatic TLC Sampler 4 (ATS4, CAMAG, Muttenz, Switzerland). The chromatographic separation was performed in the Automatic Developing Chamber 2 (ADC 2-CAMAG) with a mixture of dichloromethane and methanol (90:10 v/v), up to a migration distance of 80 mm (from the lower plate edge). The plate was pre-saturated for 5 min with the mobile phase before plate development and dried automatically for 5 min after plate development. The plate was scanned (254 and 366 nm) with the GAMAG TLC Scanner 4 and documented under UV 254 and 366 nm and after spraying with sulphuric vanillin using the TLC visualizer 2 (CAMAG). The HPTLC plate images were exported from the winCATS software (CAMAG, Muttenz, Switzerland).

### 2.6. Cell Culture

The human immortalized keratinocyte (HaCaT) cell line was obtained from the American Type Culture Collection (ATCC, Rockville, USA). Cells were maintained in Dulbecco’s modified Eagle’s medium high glucose and were supplemented with 10% fetal bovine serum, 100 U/mL penicillin and 100 μg/mL streptomycin (all from Biosera, Boussens, France). Cells were maintained in a humidified atmosphere at 37 °C, 5% CO_2_ cultured conditions. In all treatments, propolis extracts were dissolved initially in DMSO and made up to the required concentration with complete cell culture medium (final maximum concentration of DMSO 0.05% w/v). The sub-confluent cells (60–70%) were treated with either varying concentrations of propolis extracts or vehicle alone (DMSO, 0.05% (v/v) in media) that served as a control. Τreatments of cells with propolis extracts were done 2 h prior to UVB exposure and 2 h post-UVB exposure followed by 24 h recovery in culture medium. Cell viability was determined by trypan blue exclusion assay using a hemocytometer.

### 2.7. Sulforhodamine B (SRB) Assay

The cytotoxicity profile of propolis extracts was assessed by the SRB assay. Briefly, 5 × 10^3^ HaCaT cells per well were seeded in 96-well microplates, cultured for 24 h and then treated with different concentrations of propolis extracts (0–200 μg/mL) for 24 h. Then, the cells were fixed by adding 50% (w/v) cold trichloroacetic acid (TCA) (Applichem, Darmstadt, Germany) in each well and then were stained with 0.4% (w/v) sulforhodamine B (SRB) (Sigma-Aldrich, Dorset, U.K.) in 1% (v/v) acetic acid (Scharlau, Barcelona, Spain). The bound dye was dissolved in 10 mM Tris base (Sigma-Aldrich, Dorset, U.K.) and the absorbance was measured at 570 nm using a multi-plate reader (Tecan, Männedorf, Switzerland). The % cell survival was calculated using the formula:

[(Sample OD_570_ − media blank OD_570_)/(mean control OD_570_ − media blank OD570)] × 100.

The EC_50_ values (effective concentration that causes 50% decrease in cell viability) of all propolis extracts were determined by regression analysis using a four-parameter logistic curve with the Sigma Plot Software v.10 (Systat, San Jose, CA, USA).

### 2.8. Single Cell Gel Electrophoresis (Comet) Assay

The comet assay was performed as described previously with minor modifications [49]. In brief, 3 × 10^5^ HaCaT cells were seeded on 60 mm plates, cultured for 24 h, and incubated for 2 h, either with 20 μg/mL of propolis extract (diluted in culture medium) or with normal culture medium. Then, cells were either irradiated with UVB (55 mJ/cm^2^) in PBS (treated samples) by using a Bio-Link BLX254 Crosslinker (Vilber Lourmat, Marne-la-Vallée, France) or left untreated (control samples). Then, the cells were placed for 2 h in culture medium in the presence or absence of propolis samples, and then recovered for 24 h in culture medium. At the end of the incubation time the cells were collected though trypsinization, centrifuged at 1500 rpm for 2 min, washed, and resuspended in 1 × PBS (Biosera, Lewes, UK). Approximately, 2 × 10^4^ cells were suspended in 1 mL low-melting-point agarose in PBS pH 7.4 and placed onto super-frosted glass microscope slides pre-coated with a layer of 1% low-melting-point agarose. The agarose was allowed to set for 2 min at room temperature. Slides were then immersed in lysis solution (1.2 M NaCl, 100 mM Na_2_EDTA, 0.1% sodium lauryl sarcosinate, 0.26 M NaOH, pH ∼ 13) for 1 h at 4 °C, in darkness, under alkaline conditions, to allow the unwinding of DNA. Following lysis, slides were washed twice with rinse solution (0.03 M NaOH, 2 mM Na_2_EDTA, pH ~12.3) for 20 min at room temperature. Slides were subjected to electrophoresis in the rinse solution at 13 V for 25 min, neutralized in dH_2_0 and stained for 20 min with 10 μg/mL propidium iodide. Finally, they were washed with dH_2_0 and processed for observation on a Nikon ECLIPSE E200 fluorescence microscope. Image analysis and scoring of DNA damage in arbitrary units (AU) was performed as previously described [50].

### 2.9. Determination of Antioxidant Capacity in Cell Lysates

The antioxidant capacity of HaCaT cell lysates was assessed using Cayman’s antioxidant assay kit (Cayman Chemical, Ann Arbor, MI, USA) according to manufacturer’s instructions. The assay depends on the ability of antioxidants to prevent the oxidation of ABTS by metmyoglobin. The antioxidant capacity of cell lysates is compared to that of Trolox, a tocopherol analogue. In brief, 2.5 × 10^6^ HaCaT cells were seeded in 100 mm plates, cultured for 2 h in the presence or absence of propolis extracts (20 μg/mL), washed with PBS (Biosera), and then irradiated with UVB (55 mJ /cm^2^) or left untreated. Irradiated and non-irradiated cells were further incubated for 2 h with 20 μg/mL propolis extract (diluted in culture medium) or with normal culture medium, followed by 24 h recovery in culture medium. Then, the cells were collected, lysed by sonication in cold lysis buffer (5 mM potassium phosphate pH 7.4, 0.9% sodium chloride, 0.1% glucose) and centrifuged at 10,000× *g* for 15 min at 4 °C. Ten microliters (10 μL) of the supernatants were mixed with 10 μL of metmyoglobin, 150 μL of chromogen and 40 μL of 441 μM hydrogen peroxide. Absorbance was measured at 750 nm using an Enspire Multimode plate reader (Perkin Elmer, Waltham, MA, USA) and interpolated in a Trolox calibration curve. Antioxidant capacity was expressed as mM Trolox Equivalents.

### 2.10. Assessment of Protein Carbonyl Content

Protein oxidation was determined by measuring the levels of protein-bound carbonyl groups after utilizing the protein carbonyl colorimetric assay kit (Cayman Chemical). The assay relies on the reaction of protein carbonyls with 2,4-dinitrophenylhydrazine (DNPH) and subsequent detection of the produced hydrazone at 370 nm. Briefly, 2.5 × 10^6^ HaCaT cells were seeded in 100 mm plates, cultured for 2 h in the presence or absence of propolis extracts (20 μg/mL), washed with PBS (Biosera), and then were either irradiated with UVB (55 mJ/cm^2^) or left untreated. Irradiated and non-irradiated cells were incubated for 2 h either with 20 μg/mL of propolis extracts (diluted in culture medium) or with normal culture medium followed by 24 h recovery in culture medium. The cells were then collected, lysed with sonication in cold lysis buffer (50 mM MES pH 6.7, 1 mM EDTA) and centrifuged at 10,000× *g* for 15 min at 4 °C. Two hundred (200) μL of the supernatants were mixed with 800 μL DNPH (test) or 800 μL 2.5M HCl (control) and incubated, in the dark at room temperature for 1 h. Subsequently, the samples were fixed by adding 1 mL 20% TCA, incubated on ice for 5 min and then centrifuged at 10,000× *g* for 10 min at 4 °C. Pellets were washed once with 10% TCA and then three times with an ethanol/ethyl acetate mixture. Finally, protein pellets were resuspended in guanidine hydrochloride and the absorbance was measured at 370 nm using an Enspire Multimode plate reader (Perkin Elmer). The concentration of protein carbonyls was calculated using the following equation:

Conc. (nmol/mL) = [(CA)/(0.011 μM^−1^)] (500 μL/200 μL), (3)

where CA is the absorbance of the test sample (after subtracting the absorbance of the control) and was adjusted to the total protein concentration.

### 2.11. Human Reconstituted Skin Tissue Model (EpiDerm^TM^ EPI-200)

The Epiderm^TM^ EPI-200 (MaTek Inc. Ellicott City, MA, USA) is a normal, human, 3D model of skin epidermis. It consists of human-derived, normal epidermal keratinocytes cultured to reconstitute a multilayer model of epidermis. This reconstituted tissue is mitotically and metabolically active and has the capacity to mimic normal human skin epidermis. The tissue was obtained as 24-well culture plate inserts, which were then equilibrated in EPI-100 assay medium in humidified atmosphere at 37 °C, 5% CO_2_, for 24 h. Throughout the experiments, the reconstituted skin tissues were cultured in 6-well plates with the lower surface being exposed to the EPI-100 assay medium and the apical surface to air.

### 2.12. Treatment and UVB Irradiation of EpiDerm^TM^ EPI-200

The reconstituted skin tissues were topically treated, in the apical surface, with propolis extracts (20 μg/mL diluted in EPI-100 assay medium) for 2 h and then washed three times with 1 × PBS by gentle pipetting. The culture media were replaced with PBS and the skin tissues were irradiated with UVB (55 mJ/cm^2^). Following UVB irradiation, the skin tissues were topically treated again with propolis extracts for 2 h. Finally, the culture inserts with the skin tissues were placed in fresh EPI-100 assay medium and were collected 24 h post-treatment with propolis extracts for immunohistochemistry and real-time PCR analysis.

### 2.13. Quantitative Real-Time PCR

Total RNA was isolated from skin tissues with Trizol reagent (Life Technologies, Thermo Fisher Scientific, Waltham, MA, USA) according to the manufacturer’s instructions. The quality and quantity of the isolated RNA were determined spectrophotometrically and by agarose gel electrophoresis. Five μg (5 μg) of total RNA was then reverse-transcribed into cDNA using Superscript first-strand synthesis kit or RT-PCR (Life Technologies). Subsequently, quantitative real-time PCR was performed on a StepOne PCR System in MicroAmp^®^ Fast Optical 48-well reaction plates (both from Applied Biosystems, Thermo Fisher Scientific) using the KAPA SYBR^®^FAST qPCR Kit (Kapa Biosystems, Wilmington, DE, USA) under the following conditions: 95 °C for 3 min followed by 40 cycles of 95 °C for normalization. Each reaction was performed in triplicate and each experiment included two non-template controls. The sequences of *MMP1*, *MMP3*, *MMP7*, *MMP9,* and β-*actin* primers are shown in Table 2. Primer specificity was verified by melting curve analysis. For relative quantification of the transcripts, the formula RQ = 2^−ΔΔCt^ was used.

### 2.14. Immunohistochemistry (IHC)

Histopathological examination was performed on 4 mm hematoxylin–eosin stained sections and the severity of histological lesions were quantified according to the following scoring system. 0: No lesions; 1: mild lesions; 2: moderate lesions; 3: severe lesions. Lesion severity scores were added to obtain the histopathological score for all specimens.

For the detection of MMP-1, -3, -7, and -9, the reconstituted skin tissues were collected 24 h post UVB irradiation plus treatment with propolis extracts, fixed in formalin and then embedded in paraffin. Two micron (2 µm) sections were deparaffinized, rehydrated, treated with 0.3 % H_2_O_2_ for 5 min in methanol (to prevent endogenous peroxidase activity) and then were immune-stained by the peroxidase method (Envision System, DAKO, Carpinteria, CA, USA) and according to the manufacturer’s recommendations. In brief, after antigen retrieval and endogenous peroxidase blockade, the sections were blocked with Protein Block Serum-Free (DAKO) and incubated overnight at 4 °C with antibodies against MMP-1 (mouse-raised), -3 (rabbit-raised), -9 (mouse-raised) (all from Acris, Herford, Germany), and -7 (Proteintech, Machester, UK) in 1:750, 1:100, 1:900, and 1:100 dilutions respectively. Then the sections were incubated with the respective secondary antibodies at room temperature for 60 min. Finally, bound antibody complexes were stained for 10 min with 0.05% diaminobenzidine. Sections were briefly counterstained with Mayer’s hematoxylin, mounted and examined under a Nikon Eclipse 50i microscope. Control slides were incubated for the same period with non-immunized rabbit or mouse serum (negative control). Immunohistochemical antibody expression was graded in terms of the proportion of positively-stained cells after scanning the entire section of each specimen according to the following semi-quantitatively four scale scoring system (0–3): Sections with >10% stained cells were evaluated as negative (0); low (1) for 10–20%; moderate (2) for 20–50% stained cells; and high (3) for >50% positively stained cells. Intensity was not scored separately. Both histopathological and immunohistochemical evaluation was performed in a blinded fashion under a Nikon Eclipse 50i microscope by an experienced and skilled pathologist.

### 2.15. Statistical Analysis

All graphs, data statistical analyses, and calculations of EC_50_ and EC_10_ values were performed by the Graph Pad Prism 5 and Sigma Plot Software v.10 software packages. Results were expressed as mean ± SD and three independent experiments were performed in triplicate. Statistical analyses between control and treatment groups were performed using a two-tailed Student’s *t*-test. A value of *p* ≤ 0.05 was considered statistically significant.

## 3. Results

### 3.1. Chemical Characterization, Assessment of Radical Scavenging Activity, and Cytotoxicity Profile of Propolis Extracts

To investigate the potential antimutagenic, antioxidant and antiphotoaging properties of Greek propolis, ten samples of different geographic and seasonal origin were collected and subjected to extraction with a two-step sequential ultrasound-assisted method with *n*-heptane or methanol. A total of twenty (20) extracts of propolis were obtained, ten following extraction with n-heptane (PR_1a–PR_10a) and ten more following extraction with methanol (PR_1b–PR_10b). The twenty derived samples were subsequently screened for their total phenolic content and total flavonoid content (TPC and TFC, respectively). Methanolic extracts showed significant phenolic and flavonoid content (Table 3) and were further evaluated for their in vitro radical scavenging activity with the cell-free ABTS and DPPH methods. Additionally, their metabolic profiling was investigated by HPTLC.

The DPPH radical scavenging activity of methanolic propolis samples was found to exhibit versatile features, varying between 57.60% and 92.07% inhibition, while values of antiradical activity towards ABTS•^+^ were 23.83%–53.93% (Table 4). It is important to note that the samples PR_1b, PR_2b, PR_3b, PR_5b, PR_6b, PR_9b with the higher total phenolic content (TPCs ranged between 151.16 mg/g and 205.70 mg/g GAE; Table 3) showed the highest activity, demonstrating a more than 90% DPPH inhibition at 0.25 mg/mL final concentration and approximately 50% ABTS inhibition at 0.33 mg/mL. It might be claimed that it is difficult to observe direct correlations between TPCs and the radical scavenging properties of the studied propolis samples. However, in cases of both TPC and anti-scavenging activity, samples PR_7b and PR_10b, coming from the Greek islands (Chania-Crete and Samos respectively) exhibited the lowest relevant values. These results confirm previous findings of other studies on the considerable differences in terms of composition and possible biological impact of distinctive propolis samples stemming from different regions.

**Figure 1:**
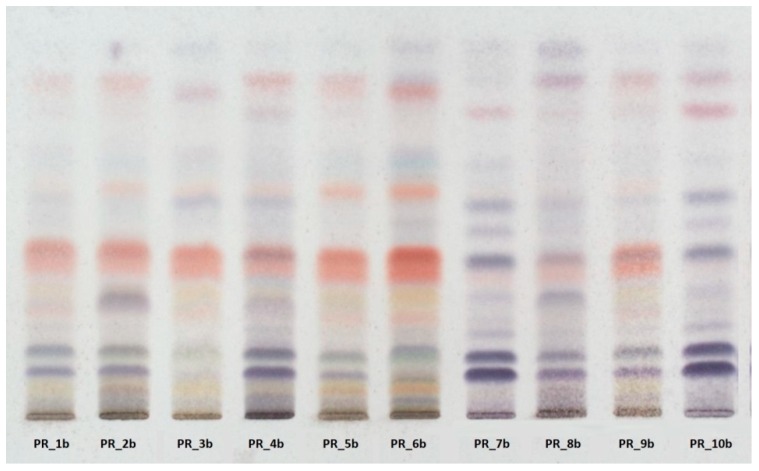
High-performance thin-layer chromatography (HPTLC) revealed high variability in the metabolic profile of the methanolic extracts.

HPTLC fingerprinting (Figure 1) also revealed that the profile of samples with the higher TPC and TFC values (PR_1b, PR_2b, PR_3b, PR_5b, PR_6b, PR_9b) is rich in flavonoid compounds, whereas the profile of samples with the lowest values (PR_7b and PR_10b) is rich in terpenoids.

Our results are in agreement with the data from the previous study of Petkova-Popova et al., as the propolis samples coming from the mainland of Greece are rich in flavonoids and phenolic acids and their esters, and therefore possess great antioxidant activity [50]. On the other hand, propolis samples coming from Greek islands belong to the Mediterranean propolis type, with a specific diterpenic profile and therefore a lower antioxidant activity [51].

**Figure 2:**
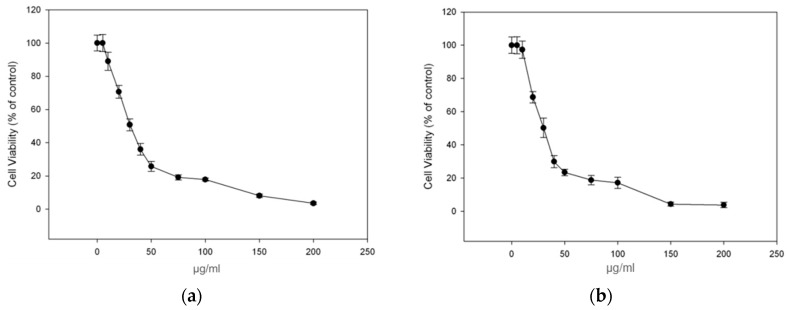
Cytotoxicity profile of propolis extracts in human immortalized keratinocyte (HaCaT) cells. Cells were incubated for 24 h with increasing concentrations (0–200 μg/mL) of each propolis extract: PR_1b (**a**) and PR_9b (**b**). Cell viability was determined by the SRB assay. The results are shown as the mean ± SD of three independent experiments.

To continue with our study, among the propolis samples with the highest antioxidant activity we selected propolis samples PR_1b and PR_9b, whose raw material were collected in large amounts for further investigation, and aimed to determine their cytotoxicity profile in the human immortalized keratinocyte (HaCaT) cell line by employing the SRB assay. Overall, the cells were treated with increasing concentration of PR_1b and PR_9b for 24 h, and cell viability was determined as percent of control. The corresponding EC_50_ and EC_10_ values (efficient concentrations that cause 50% and 10% decrease in cell viability respectively) of each propolis extract were also evaluated. As shown in Figure 2a,b, the observed patterns of cytotoxicity were very similar between all extracts with the EC_50_ and EC_10_ values ranging approximately from 26–28 μg/mL to 9–11 μg/mL respectively (Table 5). For all subsequent experiments the concentration of 20 μg/mL of propolis extracts was chosen as at the given concentration cell viability remained more than 90% following 24 h incubation with the propolis extracts compared to control.

### 3.2. Propolis Extracts Protect Human Epidermal Keratinocytes (HaCaT) Cells from UVB-Induced DNA Damage

**Figure 3:**
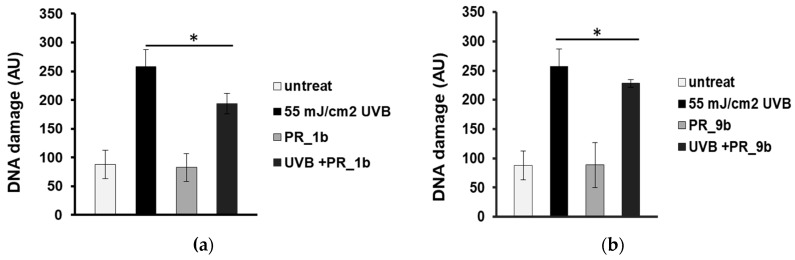
Propolis extracts protect HaCaT cells from UVB-induced DNA damage. Cells were pre-treated for 2 h with 20 μg/mL of PR_1b (**a**) and PR_9b (**b**) and either irradiated with UVB 55mJ/cm^2^ or left untreated (non-irradiated). Irradiated and non-irradiated cells were further incubated for 2 h in the presence or absence of propolis samples, recovered for 24 h in culture medium, and subjected to cell gel electrophoresis (comet) assay. The scoring was expressed in arbitrary units (AU) and was based on the extent of DNA damage under each experimental condition. The data presented are the mean ± SD of three independent experiments performed in duplicates. * *p* ≤ 0.05, significantly different from the irradiated cells.

Single cell gel electrophoresis assay (comet assay) under alkaline conditions was used to detect single- and double-strand breaks in the DNA. To determine the antioxidant and photoprotective properties of the selected propolis extracts on the formation of DNA strand breaks, HaCaT cells were pre-incubated with 20 μg/mL of PR_1b, and PR_9b and then exposed to UVB (55 mJ/cm^2^). None of the propolis extracts did cause significant increase in DNA damage levels compared to control (untreated cells) (Figure 3a,b). UVB irradiation of HaCaT cells induced significant increase in DNA damage levels which were considerably decreased in the case of cell exposure to pPR_1b (Figure 3a), and PR_9b (Figure 3b) propolis samples, suggesting promising photoprotective properties.

### 3.3. Protection of HaCaT Cells by Propolis Extracts against UVB-Induced Oxidative Damage and Photoaging

**Figure 4:**
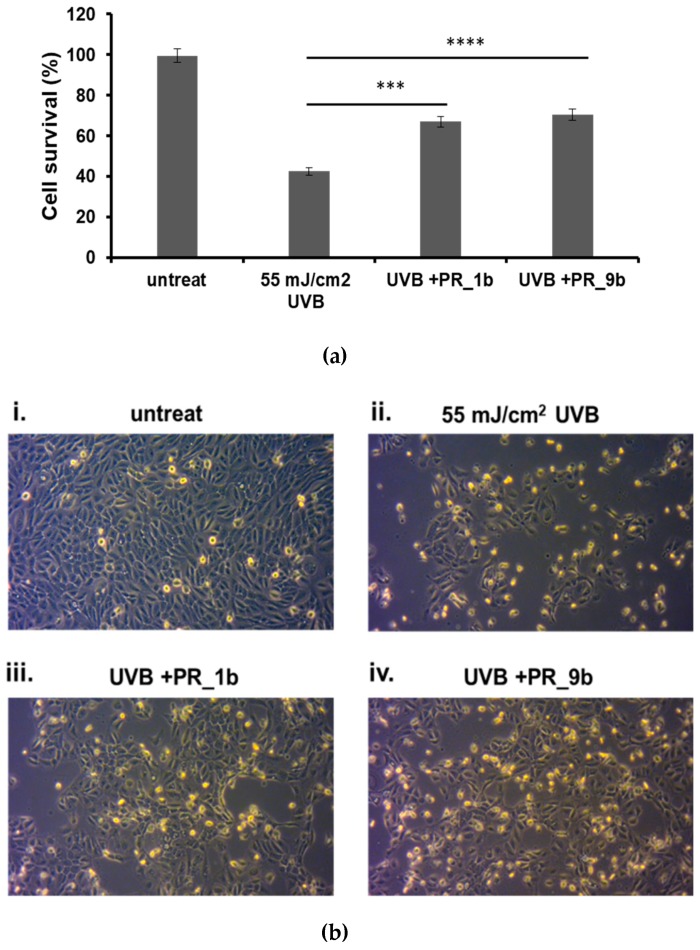
Protection of HaCaT cells by propolis extracts against UVB-induced cell cytotoxicity. Cells were pre-treated for 2 h with 20 μg/mL of propolis extracts, irradiated with 55 mJ/cm^2^ UVB and further incubated in the presence or absence of propolis extracts for 2 h. After a 24 h recovery, the remaining cell viability was assayed both through trypan blue exclusion assay (**a**) and phase contrast microscopy (10×) (**b**). (A) The data presented are the mean ± SD of three independent experiments performed in triplicate. *** *p* ≤ 0.001, **** *p* ≤ 0.0001 significantly different from the UVB-irradiated cells. (B) Phase contrast microscopy of non-irradiated (i) and UVB-irradiated (ii) cells as well as those treated with PR_1b and PR_9b (iii and iv, respectively). Figures are representative of ten random fields for each condition examined in triplicate.

Phase-contrast microscopy and the trypan blue exclusion assay were utilized for determining the effect of PR_1b and PR_9b against UVB-induced cytotoxicity in HaCaT cells (Figure 4). As noted, UVB irradiation resulted in a significant reduction of cell viability accompanied by morphological changes of HaCaT cells (Figure 4b). On the other hand, pretreatment with PR_1b and PR_9b before UVB exposure prevented the UVB-induced decrease in HaCaT cell viability (Figure 4a,b).

**Figure 5:**
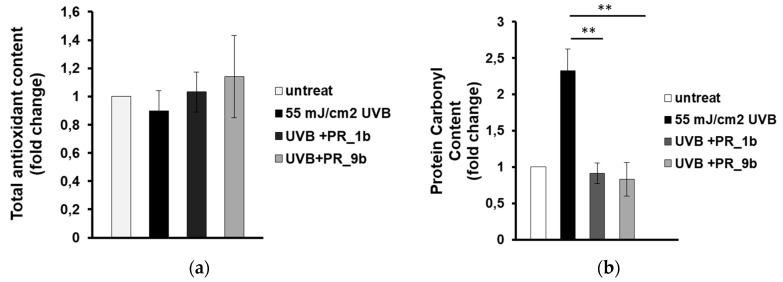
Propolis extracts protect HaCaT cells from UVB-induced oxidative protein damage. Cells were pretreated with 20 μg/mL of either PR_1b or PR_ 9b for 2 h and then exposed to UVB irradiation (55 mJ/cm^2^) or left untreated. Following UVR exposure, cells were incubated in the presence or absence of propolis extracts for 2 h followed by a 24 h recovery in culture medium before being processed for estimation of oxidative status. (**a**) Total antioxidant content and activity of cell lysates were assessed by the ABTS oxidation assay and expressed as fold change in Trolox equivalents. (**b**) Protein oxidation estimated by measuring the protein carbonyl levels with the DNPH colorimetric assay. The concentration of the protein carbonyls was determined, adjusted to the total protein concentration, and was expressessed as fold change compared to the untreated cells. Data shown are the mean ± SD of three independent experiments performed in triplicate. ** *p* ≤ 0.01, significantly different from the UVB-irradiated cells.

Next, we aimed to investigate whether the observed inhibition of UVB-induced cytotoxicity is due to the antioxidant activity of the propolis samples. HaCaT cells were treated for 2 h with either PR_1b or PR_9b prior to UVB irradiation, followed by 2 h post-treatment in the presence or absence of the propolis extracts and the antioxidant capacity was measured in cell lysates by employing the Trolox antioxidant assay. Overall, antioxidant activity levels remained statistically non-significant between untreated and UVB-irradiated cells under conditions of exposure of the cells in the absence or the presence of the two propolis samples (Figure 5). On the contrary, supplementation with either sample, PR_1b or PR_9b, under the same experimental conditions, significantly inhibited UVB-induced protein oxidation (measured as protein carbonyl content) in HaCaT cells (Figure 5b).

### 3.4. Propolis Extracts Inhibit UVB-Induced Overexpression of Matrix Metalloproteinases (MMPs) in a Human Reconstituted Skin Model

To further evaluate the protective effects of propolis extracts against UVB-induced photoaging, we employed a human reconstituted skin model known as EpidermTM EPI-200. This is a normal, human, 3D model of epidermal tissue, consisting of neonatal-derived epidermal keratinocytes that have been cultured to reconstruct a multilayer model of epidermis. It is mitotically and metabolically active and very closely mimics the human skin.

**Figure 6:**
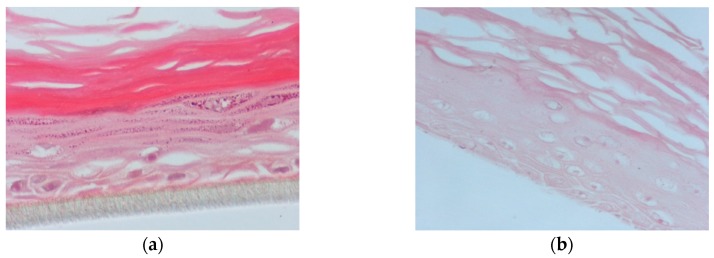

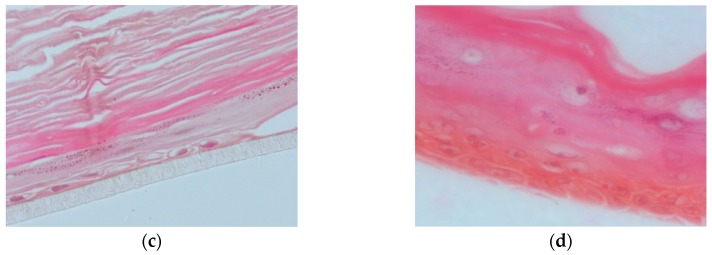
Evaluation of the protective effect of propolis extracts against UVB-induced skin damage. Epiderm^TM^ EPI-200 reconstituted human skin tissues were treated on their apical surface with 20 μg/mL of either PR_1b or PR_9b for 2 h, washed with PBS, and then exposed to 55 mJ/cm^2^ of UVB irradiation. After exposure, the apical surface of the tissues was incubated in the presence or absence of each of the propolis extracts for 2 h, then washed with PBS and placed in culture medium. After 24 h, the tissues were harvested and sections were taken. Representative figures (at 400× magnification) of eosin and hematoxylin staining of untreated tissues (**a**), UVB-irradiated skin tissues (**b**) and tissues treated with UVB and either PR_1b (**c**) or PR_9b (**d**).

In this set of experiments, the apical surface of the skin tissues was pre-treated for 2 h with PR_1b or PR_9b, exposed to 55 mJ/ cm2 UVB irradiation and post-treated with the propolis extracts for 2 h. Following 24 h, hematoxylin and eosin (H&E) staining was performed to identify potential morphological changes and skin lesions. UVB exposure caused severe cellular damage characterized by necrotic keratinocytes (Figure 6b) compared to control (Figure 6a). Supplementation of the UVB-irradiated skin tissues with PR_1b and PR_9b resulted in noticeable tissue changes including reducing keratinocyte layers and scattered sunburn cells (pyknotic nuclei) for PR_1b (Figure 6c) as well as cytoskeletal blebbing, intercellular edema, and few sunburned cells for PR_9b (Figure 6d).

**Figure 7:**
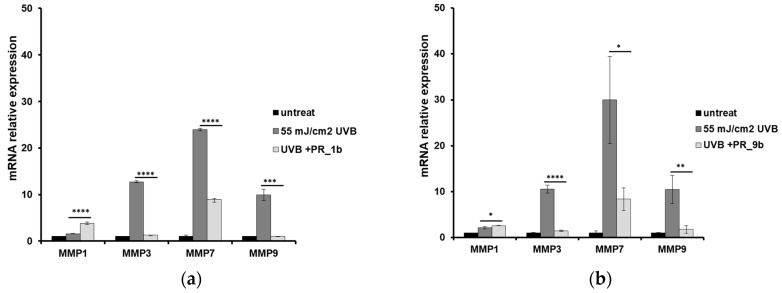
Propolis extracts decrease the mRNA levels of *MMPs* after UVB irradiation in a reconstituted human skin model. Epiderm^TM^ EPI-200 human skin tissues were pretreated with 20 μg/mL of either PR_1b (**a**) or PR_9b (**b**), for 2 h prior to UVB irradiation (55 mJ/cm^2^) and incubated again with or without the propolis extracts for 2 h. After 24 h, the tissues were harvested, and total RNA was extracted. Quantitative real-time PCR was utilized to determine levels of *MMP-1*, *-3*, *-7*, and *-9* mRNA. Expression levels of *MMP-1*, *-3*, *-7*, and *-9* were normalized to those of β-*actin*. Untreated cells served as a reference sample. The formula RQ = 2^−ΔΔCt^ was used in order to quantitate the data. The graph is representative of three independent experiments performed in triplicate. * *p* ≤ 0.05, ** *p* ≤ 0.01, *** *p* ≤ 0.001, **** *p* ≤ 0.0001.

Since induction of matrix metalloproteinases (MMPs) is a critical mechanism of photoaging, we analyzed by quantitative PCR the expression levels of *MMPs* in skin tissues that had been irradiated with UVB and then treated with either PR_1b or PR_9b (Figure 7). Significant overexpression of *MMP-1*, *-3*, *-7*, and *-9* was documented in the UVB-exposed tissues compared to the untreated skin. Incubation of the UVB-irradiated reconstituted skin with PR_1b led to a variable degree of reduction in the expression levels of *MMP-3*, *-7*, and *-9* (Figure 7a). On the other hand, a significant increase in the mRNA expression levels of *MMP-1* was noticed. Similar results were obtained in the case of the PR_9b propolis extract (Figure 7b).

**Figure 8:**
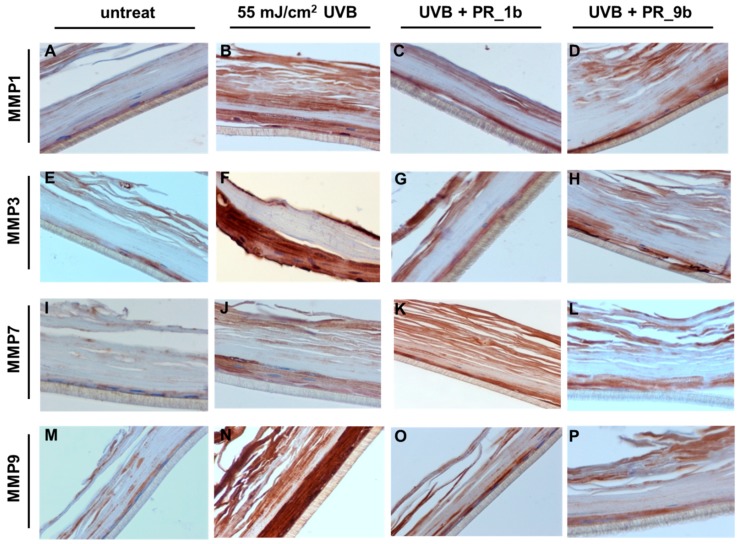
Propolis extracts protect from UVB-induced overexpression of MMPs in a reconstituted human skin model. Epiderm^TM^ EPI-200 human skin tissues were pretreated with 20 μg/mL of either PR_1b or PR_9b, for 2 h prior to UVB irradiation (55 mJ/cm^2^) and incubated again in the presence or the absence of the propolis extracts for 2 h. After 24 h recovery in culture medium, the tissues were harvested, sections were taken, and immunostaining was performed to determine the protein expression levels of MMP-1 (**A**–**D**), -3 (**E**–**H**), -7 (**I**–**L**), and -9 (**M**–**P**). Representative figures (at 400× magnification) of untreated (**A**,**I**,**J**,**M**) as well as UVB-irradiated (**B**,**F**,**J**,**N**), UVB/PR_1b (**C**,**G**,**K**,**O**) and UVB/PR_9b (**D**,**H**,**L**,**P**) treated human skin tissues (intensity scale score for staining: **A** = 1, **B** = 3, **C** = 1.5, **D** = 1.5, **E** = 1, **F** = 3, **G** = 1.5, **H** = 1.5, **I** = 1, **J** = 2, **K** = 2, **L** = 1.5, **M** = 1, **N** = 3, **O** = 2, **P** = 1.5).

To further validate the effect of propolis extracts on the UVB-induced overexpression of MMPs at protein level, immunohistochemical analysis was performed. As expected, UVB irradiation induced an upregulation of all the MMPs examined, especially MMP-3 and -9 (Figure 8B,F,J,N), compared to control (Figure 8A). In consistence with the real-time PCR data obtained from the skin tissues, treatment with PR_1b resulted in a significant decrease in the protein expression levels of MMP-3 and -9 (Figure 8C,G,O) and to a lesser extent of MMP-7 (Figure 8K). Similar results were observed with PR_9b (Figure 8D,H,L,P). Contrary to what was observed for *MMP-1* mRNA levels, both propolis extracts also resulted in a significant decrease of the UVB-upregulated MMP-1 protein, suggesting that propolis may exert regulatory functions at post-transcriptional level.

## 4. Discussion

Exposure to solar UVR is considered to be a key factor in the development of certain skin pathologies like inflammation, skin cancer, and photoaging [52,53]. UVR exhibits its strong cytotoxic effect both through direct targeting of DNA as well as through the production of reactive oxygen species (ROS) [54]. Therefore, the identification of natural compounds with a strong, multifaceted, cytoprotective effect against UVR is crucial for addressing the demand for effective, natural cosmeceutical products.

For our study, 10 propolis samples from different regions of Greece were collected and 20 propolis extracts (10 *n*-heptane and 10 methanol extracts) were produced. Initially, we analyzed the total phenolic and flavonoid content of all extracts. The methanolic extracts showed high phenolic and flavonoid content and were forwarded for the evaluation of their in vitro antioxidant capacity. Based on their in vitro antioxidant capacity the methanolic extracts appeared to be more promising. We selected the methanolic extracts PR_1b and PR_9b to continue with our study and further investigated their antioxidant and photoprotective properties. The range of the non-toxic concentration of the propolis extracts was determined in HaCaT cells and their capacity to inhibit the oxidative and DNA damaging effects of UVB was further evaluated. Finally, the antiaging properties of the extracts were determined through analyzing the effect of the propolis on the UVR-induced upregulation of MMPs in a reconstituted human skin model. Our results indicated that the selected methanolic propolis extracts exhibited significant in vitro antioxidant capacity and antimutagenic activity. Furthermore, the PR_1b and PR_9b extracts were capable of maintaining cell viability and inhibiting protein oxidation of HaCaT cells under UVB exposure conditions. Regarding the antiaging activity of the extracts, treatment with the PR_1b and PR_9b extracts protected the UVB-irradiated skin tissues from severe damage and total keratinocyte necrosis and led to reduced UVB-dependent upregulation of the *MMP-3*, *MMP-7,* and *MMP-9* genes as well as lower MMP-1, MMP-3, and MMP-9 protein levels in comparison to the untreated UVB-irradiated skin tissues.

Even though the comparison between extracts produced by propolis samples from different geographic regions is particularly complex as a result of the significant differences in their chemical compositions due to the variability of the local flora on the collection sites, in general, our results are in line with previous experimental data supporting the protective properties of propolis. Specifically, the antioxidant capacity of propolis extracts is considered to be well established, specifically through commonly used in vitro antioxidant methods. In fact, in most cases the antioxidant activity of the extracts appeared to have a direct correlation with their total phenolic and/or total flavonoid content [55,56,57,58,59,60]. In our study, all the examined methanolic extracts of propolis exhibited comparable in vitro antioxidant activity (23.83% to 53.93% and 57.60% to 92.07% for ABTS and DPPH, respectively) regardless of the range of their total phenolic (55.67 to 205.70 mg GAE/g dry extract) and/or flavonoid (54.02 to 215.76 mg QE/g dry extract) content. This finding could be explained by the fact that while flavonoid and phenolic compounds are major constituents of propolis, it also consists of a huge variety of additional chemical compounds, some of which possess certain antioxidant properties [61].

Additionally, our results showed that all the examined methanolic extracts demonstrated protective effects against the DNA-damaging potential of UVB radiation. Our data agree with previous reports describing the antimutagenic activity of propolis extracts [62,63,64,65,66]. For instance, Roberto et al. reported that treatment with ethanolic extracts of green type Brazilian propolis significantly reduced the methyl methanesulfonate (MMS)-induced DNA damage in the rat hepatoma cell line HTC [63], while Yalcin et al. demonstrated that pre-treatment with ethanolic extract of Turkish propolis substantially reduced the γ-ray-induced DNA damage of foreskin fibroblast cells [64]. Similarly, Benkovic et al. showed that pre-treatment with ethanolic propolis extracts was efficient to protect human white blood cells from the genotoxic effects of γ-radiation, while administration of ethanolic propolis extracts to mice prior to their irradiation with γ-rays resulted in reduced DNA damage levels of their white blood cells and in higher survival rates [65,66]. To the extent of our knowledge, this is the first time that the antimutagenic activity of propolis extracts against UVR is being demonstrated in human keratinocyte HaCaT cells.

Among the propolis samples, PR_1b and PR_9b were selected for further investigation. In our study, treatment with the selected propolis extracts PR_1b and PR_9b protected HaCaT cells from the UVB-induced protein oxidation and cytotoxicity. Our findings are supported by previous data from Kim et al. who reported that treatment with propolis led to inhibition of UVA-induced apoptosis and caspase-3 activation accompanied by attenuated UVA-dependent ROS formation in HaCaT cells [67]. Similarly, Fonseca et al. demonstrated that the oral administration of propolis extracts to hairless mice prevented the UV-induced depletion of glutathione (GSH) [68], while Bolfa et al. showed that topical treatment of propolis extracts to mice reduced the UVB-induced generation of malondialdehyde and re-established glutathione peroxidase activity [31].

Finally, only a few data are currently available in the literature on the antiaging properties of propolis. For instance, Ebadi et al. reported that pre-treatment of human dermal fibroblasts with propolis extracts prior to UVB exposure resulted in increased cell viability and reduced numbers of senescent, β-galactosidase-positive cells and led to upregulation of the *FOXO3A* and *NGF* genes in comparison to the untreated dermal fibroblasts [69]. Regarding the ability of propolis to down-regulate the expression of certain MMPs, Jin et al. showed that propolis extracts significantly inhibited the MMP-9 activity in hepatocarcinoma cells [70], while Saavedra et al. demonstrated that treatment of activated macrophage cells with ethanolic propolis extracts downregulated the expression of MMP-9 in a dose-dependent manner [71]. However, these studies were focused on the antitumoral and anti-inflammatory properties of propolis and consequently were not conducted with skin cell models. MMPs are broad substrate specificity endopeptidases mediating the degradation of different components of the extracellular matrix (ECM) involved in various pathophysiological processes including, inflammation, cancer, would healing and photoaging. In skin, MMPs are secreted by keratinocytes and dermal fibroblasts in response to various stimuli including cytokines, oxidative stress, and UVR. [72]. Although the role of MMPs expressed in dermis is well characterized in relation to photoaging and wrinkle formation as they degrade ECM, less is known for the role of MMPs in keratinocytes as UVB sensors. Other studies, as well, have documented the induction of MMP-9 [73], MMP-1 [74] and MMPs-13, 12, 3, 10 [75] as a result of UVB irradiation suggesting that their up-regulation appears to be a direct effect of UVB irradiation, thus potentially contributing to photoaging through modulating apoptotic and inflammatory responses involved in skin photodamage. However, the detailed mechanisms by which MMPs in keratinocytes mediate tissue damage require further elucidation. To the extent of our knowledge, this is the first time that the inhibitory effect of propolis extracts on the UV-induced upregulation of a panel of aging-related MMPs is reported both in HaCaT cells and further validated in a reconstituted human skin model suggesting a novel role of propolis as a protective means against UVB-induced skin photodamage.

## 5. Conclusions

Τhe results of this study support the conclusion that Greek propolis extracts possess strong antioxidant, antimutagenic, and antiaging properties, and can protect keratinocytes from the multifaceted deleterious effects of UVR. Thus, they could be considered as promising candidates for the development of novel and highly effective cosmeceuticals. Future research is required though to further validate the biological activities of Greek propolis extracts, define in detail the underlying molecular mechanism(s) of action and link their cytoprotective actions with the structural elucidation of their active ingredients.

## Figures and Tables

**Table 1:** Geographical origin and season of harvest of propolis samples used in the study.

Propolis Sample	Origin	Season of Harvest
PR_1	Arta Ano Peta	Fall 2013
PR_2	Arta Mainland	Fall 2013
PR_3	Mt Olympus	Fall 2013
PR_4	Drama	Fall 2013
PR_5	Arta Mountainous	Fall 2013
PR_6	Serres	Fall 2013
PR_7	Chania-Stavros	Fall 2013
PR_8	Evia	Fall 2013
PR_9	Mt Olympus	Spring 2013

**Table 2:** The primers used for real-time PCR.

Gene	Forward Primer (5′→3′)	Reverse Primer (5′→3′)
*MMP-1*	CCTCGCTGGGAGCAAACA	TTGGCAAATCTGGCGTGTAA
*MMP-3*	GAGGCATCCACACCCTAGGTT	ATCAGAAATGGCTGCATCGAT
*MMP-7*	CTGCATTTCAGGAAAGTTGTATGG	AGCTCCTCGCGCAAAGC
*MMP-9*	GGACGATGCCTGCAACGT	CAAATACAGCTGGTTCCCAATCT
*β-actin*	GCGCGGCTACAGCTTCA	CTTAATGTCACGCACGATTTCC

**Table 3:** Total phenolic content (TPC) and total flavonoid content (TFC) of the ten extracts of n-heptane (1a–10a) and the ten methanolic propolis extracts (extracts 1b–10b) examined in the present study. Total phenolic and flavonoid contents are expressed as mg gallic acid and quercetin equivalents (GAE and QE), respectively, per gram of dry extract.

Propolis Extract	TPC C = 0.1 mg/mL mg GAE/g Extract	TFC C = 0.1 mg/mL mg QE/g Extract
PR_1a	6.54	14.28
PR_2a	8.87	14.81
PR_3a	8.36	17.09
PR_4a	8.30	19.48
PR_5a	9.29	15.09
PR_6a	10.93	16.78
PR_7a	6.49	6.80
PR_8a	5.37	9.98
PR_9a	10.18	13.45
PR_10a	10.72	11.99
PR_1b	160.64	158.82
PR_2b	151.16	181.43
PR_3b	189.44	155.91
PR_4b	107.73	101.51
PR_5b	205.70	215.76
PR_6b	154.51	134.88
PR_7b	55.67	54.02
PR_8b	93.90	75.09
PR_9b	172.24	169.03
PR_10b	74.79	58.64

**Table 4:** ABTS and DPPH free radical activity of the ten methanolic propolis extracts examined in the present study.

Propolis Extract	ABTS Inhibition (%) (C = 0.33 mg/mL)	DPPH Inhibition (%) (C = 0.25 mg/mL)
PR_1b	50.60	91.79
PR_2b	44.90	91.93
PR_3b	48.41	92.07
PR_4b	32.44	88.62
PR_5b	53.93	91.47
PR_6b	44.80	90.36
PR_7b	31.11	57.60
PR_8b	27.11	83.90
PR_9b	45.37	90.67
PR_10b	23.83	62.99

**Table 5:** The EC_50_ and EC_10_ values of the propolis extracts PR_1b and PR_9b.

Propolis Extract	EC_50_ (μg/mL) ^1^	EC_10_ (μg/mL) ^1^
PR_1b	57.34 ± 3.45	22.18 ± 0.87
PR_9b	69.30 ± 2.76	23.32 ± 0.76

^1^ Determined from the dose-response curves of Figure 1. The results are shown as the mean ± SD of three independent experiments.
